# Safety of ustekinumab in adolescent patients with moderate‐to‐severe plaque psoriasis: real‐world evidence from an ongoing European study (NCT03218488)

**DOI:** 10.1111/jdv.18110

**Published:** 2022-04-06

**Authors:** E. Mahé, A. Geldhof, M. Jazra, P. Bergmans, A. Azzabi, M.M.B. Seyger

**Affiliations:** ^1^ Department of Dermatology Centre Hospitalier Victor Dupouy Argenteuil France; ^2^ Medical Affairs Janssen Biologics BV Leiden Netherlands; ^3^ Medical Affairs Janssen‐Cilag Paris France; ^4^ Biostatistics Janssen‐Cilag BV Breda Netherlands; ^5^ Medical Affairs Janssen Near East, Maghreb & Africa Casablanca Morocco; ^6^ Department of Dermatology Radboud University Medical Center Nijmegen Netherlands

## Conflicts of interest

E. Mahé has undertaken paid activities as consultant, advisor or speaker for AbbVie, Amgen, Celgene, Janssen, Leo Pharma, Lilly and Novartis. A. Geldhof, M. Jazra, P. Bergmans and A. Azzabi are employees of Janssen and may own company stock/stock options. M.M.B. Seyger received grants from/was involved in clinical trials with AbbVie, Amgen, Celgene, Eli Lilly, Janssen, Leo Pharma and Pfizer; and served as a consultant for AbbVie, Eli Lilly, Janssen, Leo Pharma, Novartis, Pfizer and UCB, with fees paid directly to her institution.

## Ethical approval

This study complied with ethics requirements as specified by the Independent Ethics Committee/Institutional Review Board and by local regulations. Each participant signed a participation agreement/informed consent form in line with local regulations and trial sponsor policy before data collection.

*Dear Editor,*

The European Medicines Agency approved ustekinumab for treating adolescent psoriasis patients in 2015,^1^ following the phase 3 CADMUS trial.^2^ However, long‐term safety and efficacy data in this population are lacking.^3^, ^4^, ^5^ We present real‐world safety data for ustekinumab in adolescents with moderate‐to‐severe psoriasis.

This 42‐month progress report of a real‐world, postapproval study (NCT03218488) comprised 70 patients enrolled between 25 October 2017 and the progress report data cut‐off (8 January 2021). Included patients were ≥12 to <18 years of age with a confirmed diagnosis of moderate‐to‐severe psoriasis who were initiated on ustekinumab treatment within 3 months before, or 2 months after, the first assessment. Patients enrolled in an interventional clinical trial were excluded. After baseline data collection, follow‐up visits were conducted every 3 months for the first year and at least every 6 months thereafter, for a maximum of 6 years or until the patient reached 18 years of age, early withdrawal or study closure. Serious infections, malignancies and adverse events related to autoimmunity were predefined serious adverse events of special interest (SAESI).

Most patients (76.8%) started ustekinumab at a dose of 45 mg (0.30–1.41 mg/kg); 12 patients (17.1%) required a dose increase (reportedly to improve efficacy, *n* = 6; due to weight increase, *n* = 4; to improve both efficacy and safety, *n* = 1; for other reasons, *n* = 1). Nine patients (12.9%) stopped or interrupted ustekinumab therapy due to lack of efficacy (*n* = 2), lack of efficacy and adverse events (AEs; arthralgia and appendicitis, *n* = 2), AEs (asthenia and superinfected plantar psoriasis, *n* = 2) or did not attend further visits (*n* = 3).

A total of 184 AEs were reported by 39 patients (55.7%), with headache (*n* = 11, 15.7%), fatigue (*n* = 10, 14.3%) and pyrexia (*n* = 8, 11.4%) being the most frequently reported. Table 1 lists AE characteristics.

**Table 1:** Adverse event characteristics, as reported by investigator

	Overall
	N = 70
Patients with any AE	39 (55.7)
Number of events	184
Severity^a^	
Mild	100 (54.3)
Moderate	68 (37.0)
Severe	16 (8.7)
Action taken with ustekinumab^a^	
Dose increased	1 (0.5)
Dose not changed	165 (89.7)
Dose reduced	0 (0.0)
Drug interrupted	5 (2.7)
Drug withdrawn	3 (1.6)
Not applicable^b^	10 (5.4)
Unknown	0 (0.0)
Concomitant therapy given for the AE^a^	
Yes	58 (31.5)
No	121 (65.8)
Not applicable^b^	3 (1.6)
Unknown	2 (1.1)
Relationship with ustekinumab^a^	
Not related	67 (36.4)
Doubtful	45 (24.5)
Possible	57 (31.0)
Probable	3 (1.6)
Very likely	12 (6.5)
Outcome^a^	
Fatal	0 (0.0)
Not recovered/resolved	36 (19.6)
Recovered/resolved with sequelae	0 (0.0)
Recovered/resolved	130 (70.7)
Recovering/resolving	12 (6.5)
Unknown	6 (3.3)

Data are n (%) unless otherwise stated.

AE, adverse event.

^a^ Percentages are calculated from the total number of AEs with known data (excluding missing data).

^b^ ‘Not applicable’ was selected when the start date/start time of the AE was either during the screening and prior to the first injection or was after discontinuation of study drug and did not lead to discontinuation.

**Figure 1:**
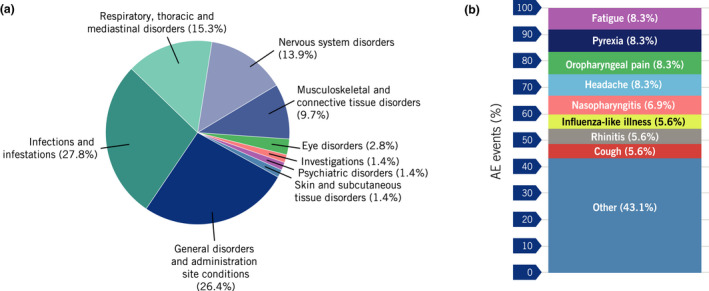
Percentage of AEs related to ustekinumab^a^ treatment (*n* = 72) by (a) system organ class and by (b) preferred term. AEs designated as ‘Other’ had ≤3 events related to ustekinumab. ^a^An AE related to ustekinumab is defined as an AE with a possible, probable or very likely relationship to ustekinumab. AE, adverse event.

Overall, 72 treatment‐related AEs were reported in 20 (28.6%) patients (Figure 1). Fatigue, pyrexia, oropharyngeal pain and headache were the most frequent, with each being reported in six patients (8.6%); all represent previously designated adverse drug reactions for ustekinumab.^6^

Thirteen serious AEs (SAEs) were reported in nine patients (12.9%), of which two were considered of special interest. The first SAESI was a case of appendicitis (serious infection) that was considered possibly related to ustekinumab; it was classified as severe and resulted in the interruption of ustekinumab treatment. The second SAESI was a case of severe worsening of pre‐existing plaque psoriasis (due to the relationship between plaque psoriasis worsening and autoimmunity), considered unrelated to ustekinumab. Both SAESIs resolved. The remaining SAEs were considered unrelated to ustekinumab treatment. No cases of malignancy or death were reported.

The percentage of patients reporting at least one AE during ustekinumab treatment was lower in this study compared with the similar population in the CADMUS trial (55.7% vs 89.3%). This may reflect more limited recall of AE‐related information at patient visits which, for this study and the CADMUS trial, were every 6 and 3 months, respectively. This may have led to less complete capture of information, underreporting of AEs, or introduction of additional biases.^2^ The relatively small sample size may reflect challenges in patient enrolment due to the relatively low prevalence of moderate‐to‐severe psoriasis among adolescents, the limited number of patients eligible for biologic therapy, restrictions in access due to local reimbursement policies and self‐isolation, quarantine and travel restrictions related to the COVID‐19 pandemic.^7^

In conclusion, this study identified no new safety concerns related to ustekinumab treatment in adolescent patients, with findings being generally consistent with the known safety profile for ustekinumab. After study completion in 2032, the results will provide a better understanding of the long‐term efficacy and safety profile of ustekinumab in adolescents with moderate‐to‐severe psoriasis.

## Funding sources

This article was sponsored by Janssen. Medical writing and editorial support were funded by Janssen.

## Data Availability

These data are not currently available for sharing. Requests for sharing will be evaluated on an individual basis.
